# Evolution of Framingham cardiovascular risk score in HIV-infected patients initiating EFV- and LPV/r-based HAART in a Latin American cohort

**DOI:** 10.7448/IAS.17.4.19547

**Published:** 2014-11-02

**Authors:** Diego Cecchini, Maria Ines Mattioli, Julia Cassetti, Debora Chan, Isabel Cassetti

**Affiliations:** 1 Helios Salud, Area Medica, Buenos Aires, Argentina; 2 Instituto de Desarrollo Humano, Universidad Nacional de General Sarmiento, Buenos Aires, Argentina; 3 Facultad Regional Buenos Aires, Departamento de Matemática, Universidad Tecnológica Nacional, Buenos Aires, Argentina

## Abstract

**Introduction:**

Epidemiological studies suggest that some antiretroviral drugs may contribute to increase cardiovascular risk in HIV-infected patients. However, data from Latin American countries are limited, as impact of HAART on cardiovascular risk remains understudied. In this context, we aimed to evaluate if 10-year Framingham Cardiovascular Risk Score (FCRS) increases in patients following exposure to EFV- and LPV/r-based HAART in a Latin American cohort.

**Materials and Methods:**

Retrospective 48-week cohort study. We reviewed clinical charts of randomly selected samples of patients initiating (according to national guidelines) EFV first-line HAART and LPV/r first- or second-line (but first PI-based) HAART assisted at a reference HIV centre in Buenos Aires, Argentina (period 2004–2012). Each patient could only be included in one arm. FCRS was calculated according to National Institutes of Health risk assessment tool (http://cvdrisk.nhlbi.nih.gov/).

**Results:**

A total of 357 patients were included: 249 in EFV arm and 108 in LPV/r arm (80 as first line and 28 as second line, but first PI-based HAART). Baseline characteristics (median, interquartile range): age, 38 (33–45) years; male, 247 (69%); viral load, 98200 (20550–306000) copies/mL; CD4 T-cell count, 115 (60–175) cel/µL; total cholesterol, 159 (135–194) mg/dL; HDL: 39 (31–41) mg/dL; LDL: 94 (72–123) mg/dL; current smoker, 29%; on antihypertensive drugs: 14 (4%), diabetic: 4 (1%). Most frequent accompanying nucleoside reverse transcriptase inhibitors (NRTIs) were 3TC (92%) and zidovudine (AZT; 76%). Baseline FCRS was low, moderate and high for 93%, 7% and 0% of patients on EFV arm and 96.7%, 1.7% and 1.7% on LPV/r arm. On EFV arm, an increase in FCRS category (low to moderate or moderate to high) was observed in 1 patient (0.9%) at 24 weeks and 6 (5,6%) at 48 weeks; 5 (4.7%) decreased category. On LPV/r arm no one varied FCRS category at 24 weeks and 2 (3.4%) increased from low to moderate at 48 weeks (no patient decreased FCRS category). Cumulative incidence of overall cardiovascular events was 1.6% on EFV and 1.8% on LPV/r arms respectively. Probability of increasing FCRS category or having a cardiovascular event did not differ between arms at a significance level of 5%.

**Conclusions:**

Probability of increasing FCRS category and cardiovascular events was low and similar in patients exposed to EFV versus LPV/r-based HAART in a Latin American cohort. ClinicalTrials.gov Identifier: NCT01705873.
